# Deciphering the impact of sepsis phenotypes on improving clinical outcome predictions: a multicenter retrospective analysis based on critical care in China

**DOI:** 10.1038/s41598-025-93961-y

**Published:** 2025-04-08

**Authors:** Luyao Zhou, Weimin Zhang, Min Shao, Cui Wang, Yu Wang

**Affiliations:** 1 School of Biomedical Engineering, Anhui Medical University, Hefei, 230032 China; 2 Department of Critical Care Medicine, First Affiliated Hospital of Anhui Medical University, Hefei, China

**Keywords:** Sepsis, Clustering algorithms, Machine learning, Intensive care, Phenotypes, Diagnostic markers, Experimental models of disease

## Abstract

Sepsis is a clinically heterogeneous disease with high mortality. It is crucial to develop relevant therapeutic strategies for different sepsis phenotypes, but the impact of phenotypes on patients’ clinical outcomes is unclear. This study aimed to identify potential sepsis phenotypes using readily available clinical parameters and assess their predictive value for 28-day clinical outcomes by logistic regression analysis. In this retrospective analysis, researchers extracted clinical data from adult patients admitted to the First Affiliated Hospital of Anhui Medical University between April and August 2022 and from the 2014–2015 eICU Collaborative Study database. K-Means clustering was utilized to identify and refine sepsis phenotypes, and their predictive performance was subsequently evaluated. Logistic regression models were trained independently for each phenotype and five-fold cross-validation was used to predict clinical outcomes. Predictive accuracy was then compared to traditional non-clustered prediction methods using model assessment scores. The study cohort consisted of 250 patients from the First Affiliated Hospital of Anhui Medical University, allocated in a 7:3 ratio for training and testing, respectively, and an external validation cohort of 3100 patients from the eICU Cooperative Research Database. The results of the phenotype-based prediction model demonstrated an improvement in F1 score from 0.74 to 0.82 and AUC from 0.74(95%CI 0.71–0.80) to 0.84(95%CI 0.82–0.87), and these results also highlight the superiority of clinical outcome prediction with the help of sepsis phenotypes over traditional prediction methods. Phenotype-based prediction of 28-day clinical outcomes in sepsis demonstrated significant advantages over traditional models, highlighting the impact of phenotype-driven modeling on clinical outcomes in sepsis.

## Introduction

Sepsis is a common and highly lethal clinically heterogeneous disease caused by invading pathogenic microorganisms, such as bacteria, into the organism, causing a systemic inflammatory response syndrome. The Third International Consensus Conference notes that sepsis can further progress to severe sepsis and septic shock, depending on the severity^1–5^. Globally, there are more than 19 million new cases of sepsis each year, with a mortality rate of more than 30%. Of the surviving patients, approximately 3 million have cognitive dysfunction^6,7^. The morbidity and mortality of sepsis are exceptionally high in developing or less developed countries^8^.

The high mortality rate of sepsis raised concerns, and accurate prediction of patients’ 28-day clinical outcomes was essential to improve patient prognosis and survival. However, many current studies did not adequately consider the differences between patients with varying severity levels. In 2020, Guilan Kong et al. utilized the MIMIC-III database to predict patients’ clinical outcomes using four models compared with simplified acute physiology score (SAPS)^9^. In 2022, C.Bao et al. used the MIMIC-IV and eICU Collaborative Research Database(eICU) databases to predict clinical outcomes in sepsis using seven machine learning methods^10^. Both studies focused on clinical outcome prediction in sepsis patients rather than patient typing. Considering the clinical heterogeneity of sepsis, patient typing followed by 28-day clinical outcome prediction could lead to more accurate results. However, one of the biggest obstacles to current sepsis staging was its overly broad definition, which led to different combinations of features producing different results.

Although Sequential Organ Failure Assessment (SOFA) scores and vital signs were commonly used for typing in clinical practice, their lack of specificity usually led to delays in treating patients^3,11,12^. In 2022, Zhenxing Xu et al. collected SOFA scores to predict phenotypes using databases such as MIMIC-III and eICU and analyzed mortality predictions. The results validated four new clinically defined phenotypes^13^. Furthermore, in 2022, Sivasubramanium V. Bhavani implemented a phenotypic classification of sepsis using the MIMIC database to capture information about features related to vital signs, and the clustering results identified four life-trajectory phenotypes^14^. In addition to the use of SOFA scores, fewer studies have used variables such as matrix metalloproteinase 8 (MMP8) and proteinase 3 (PRTN3)^15^. However, these marker assays were expensive, and some features, such as SOFA, involved subjective elements, limiting their ability to feature the phenotype fully.

While numerous studies have verified the clinical factors linked to sepsis mortality, the unique nature of sepsis means that not all patients can be considered uniform in their presentation. In summary, previous studies on phenotype-driven sepsis classification have largely been confined to single datasets such as MIMIC-III, which limits the generalizability of the developed models. Furthermore, although some studies have identified differences in mortality rates across phenotypes, they have not performed predictive analyses to compare clinical outcomes in sepsis based on phenotypic classification. Therefore, typing models must be constructed using common biological indicators to more accurately predict clinical outcomes. The contributions of this study included:

1. Basic vital signs and laboratory values measured within the first 24 h of admission were collected from several hospitals in Anhui Province. The inclusion of this multicenter dataset effectively mitigated the limitations inherent in using a single dataset. A sepsis phenotyping study was conducted using the K-Means clustering algorithm, which identified three phenotypic categories based on 12 key features.
2. Based on the clustering results, a logistic regression model for each category was constructed. Compared to the model constructed from unclassified data, the AUC value for predicting clinical outcomes 28 days after classification improved to 0.84.
3. Based on the differential impact of clustered phenotypes on clinical outcome prediction, this study accurately identified patients with distinct phenotypes to propose targeted therapeutic regimens. High-risk patients were prioritized for medical resources, including early anti-inflammatory treatments and comprehensive organ supportive care. These strategies offer novel insights and methodologies for sepsis staging and clinical outcome prediction.

## Materials and methods

The experimental procedure was divided into several key steps. Initially, the clinical features of the subjects were determined based on relevant literature and input from medical experts, and missing values were addressed through multiple imputation techniques. Subsequently, the processed data underwent clustering analysis using the K-Means algorithm. Distinct classifiers were then trained for each patient category to predict clinical outcomes. Finally, the accuracy of predicting clinical outcomes post-clustering was evaluated by comparing it with a model built using a traditional non-clustered dataset. The model’s generalizability was demonstrated using an external validation dataset from the eICU.

### Study design

**Fig. 1:**
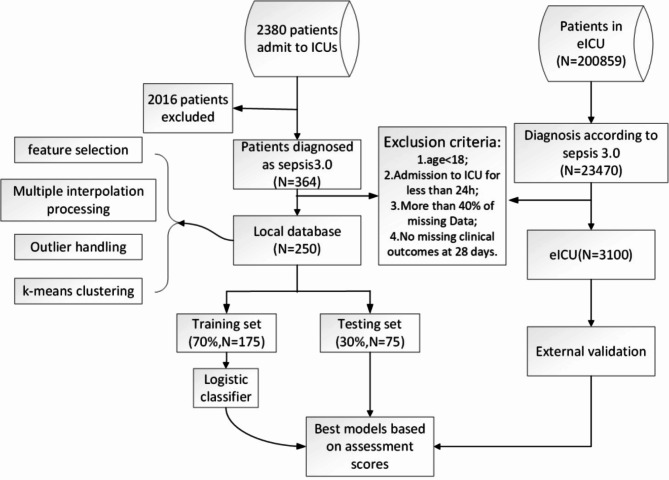
Study-specific flowchart. The figure illustrates the entire process of training the model using internal and external validation datasets after inclusion in the analysis according to the exclusion criteria.

Based on the two major criteria for the diagnosis of sepsis proposed by the Third International Consensus Conference on Sepsis in 2016 (confirmed infection or suspected infection, and Sequential Organ Failure Assessment (SOFA) score > = 2^3^), in this study, 250 sepsis patients from the Intensive Care Unit of the First Affiliated Hospital of Anhui Medical University was collected as an internal validation dataset, including 125 dead patients and 125 surviving patients. The eICU database, which provides multicenter external validation, contains more than 139,000 hospitalizations from 2014 to 2015 inclusive, with a total of 3,100 data points being collected for inclusion in the test set^16^. The inclusion criteria for internal validation and external validation were as follows: (1) adult patients 18 years of age or older; (2) hospitalization in the ICU for more than 24 h with sufficient data; and (3) patients with confirmed or suspected infection according to the Third International Consensus Definition of Sepsis (Sepsis-3) accompanied by a Sequential Organ Failure Assessment (SOFA) score > = 2 in the first 24 h after ICU admission. The process framework of the research design is shown in Fig. 1.

### Data preprocessing and variable selection

The study extracted a set of clinical variables from a local database, which included common parameters such as demographic variables (e.g., age, gender, height, weight, etc.), laboratory values (e.g., albumin, platelets, partial pressure of oxygen, partial pressure of carbon dioxide, leukocytes, erythrocytes, blood urea nitrogen, C-reactive protein, serum chloride, serum creatinine, serum sodium, serum potassium, serum fibrinogen, etc.), vital signs (e.g., heart rate, systolic blood pressure, diastolic blood pressure, mean arterial pressure, respiratory rate, body temperature, and peripheral capillary oxygen saturation), and medication modalities (e.g., mechanical ventilation, use of vasoactive drugs, and blood purification). For each variable incorporated into the model, clinical data extracted from the initial record within 24 h before admission to the intensive care unit were utilized, and any missing values were handled through multiple imputation. In the feature selection section, variables with missing values exceeding 40% were first eliminated, and highly correlated variables among the pre-existing covariates were also removed. The pre-selected variables were sourced from previously published literature and studies related to sepsis clustering^17–19^. In addition to drawing on features commonly utilized in prior sepsis typing studies, this study also undertook a preliminary exploratory analysis of clinical features. Through correlation and statistical analyses, we identified features associated with clinical outcomes. Ultimately, 12 variables were selected for the K-Means clustering algorithm following a consensus reached among four clinical critical care medicine experts. The 12 features included in the model were heart rate (HR), systolic blood pressure (SBP), respiratory rate (Breath), body temperature (TEMP), sodium (NA), hemoglobin (HB), blood urea nitrogen (BUN), C-reactive protein (CRP), oxygenation index (PaO2/FiO2), white blood cells (WBC), serum fibrinogen (FIB), and total bilirubin (TBIL).

HR: Heart rate measured at the patient’s first ICU admission.

SBP: Systolic blood pressure values measured at the patient’s first ICU admission.

Breath: Respiratory rate measured at the patient’s first ICU admission.

TEMP: The patient’s temperature was measured on his first ICU admission.

NA: Sodium values measured at the patient’s first ICU admission.

HB: Hemoglobin measured on the patient’s first ICU admission.

BUN: Blood Urea Nitrogen measured on the patient’s first ICU admission.

CRP: C-reactive protein measured on the patient’s first ICU admission.

PaO2/FiO2: The oxygenation index, measured at the patient’s initial ICU admission, was calculated by dividing the partial pressure of arterial oxygen (PaO2) by the fraction of inspired oxygen (FiO2).

WBC: Leukocytes measured on the patient’s first ICU admission.

FIB: Fibrinogen measured on the patient’s first ICU admission.

TBIL: Total bilirubin measured on the patient’s first ICU admission.

### Phenotype typing

This study used the K-means clustering algorithm to determine the number of clusters by first assessing the correlation between the variable features. A high correlation was considered, and where appropriate, the chi-square test value of their features was evaluated to determine the specific variables to be included in the model. The variables were subjected to unsupervised clustering using the K-means algorithm. This study applied K-means clustering to patients using both local and eICU datasets. The clustering results were assessed using the Silhouette score (ranging from − 1 to 1, with values closer to 1 indicating better clustering), the Calinski-Harabasz score (with higher values indicating better clustering), and the Davies-Bouldin score (Values closer to 0 indicate better clustering). Additionally, the usability of these clustering results was evaluated, and the clustering results were reproduced using the Gaussian Mixture Model (GMM). Subsequently, Principal Component Analysis (PCA) was employed to visualize these groups.

### Model selection

Multivariate logistic regression algorithms were used to estimate the correlation between sepsis phenotypes and patient clinical outcomes, comparing this with the direct effect of sepsis phenotypes on mortality in unstratified data. Model 1 predicted 28-day outcomes for patients using logistic regression based on categorical phenotypes. Model 2, on the other hand, conducted a predictive analysis of mortality based on clinical characteristics for all patients without phenotyping.

### Ethical approval

The project obtained ethical approval from the Medical Ethics Committee of the First Affiliated Hospital of Anhui Medical University (Approval No. PJ2022-01-09). In addition, data from the eICU was used. Human subject training was conducted through the CITI program (Record ID: 11706576), and this study was deemed exempt from patient informed consent and ethical approval^20^.

## Result

### Queue features

As detailed in Sect. 2, the dataset consisted of 12 features and included 250 patients with sepsis. The study enrolled all adult (≥ 18 years) sepsis patients, retaining data only within the first 24 h of admission for those with multiple admissions. To ensure data quality, patients with more than 40% missing values were excluded following data validation. Tables 1, 2 provide a detailed breakdown of the clinical characteristics of patients in the local and eICU datasets. The local dataset had a median age of 69 years (range 18–90), with 61.2% male and all patients being Asian. In contrast, the eICU dataset had a median age of 64 years (range 18–90), with 54% male and the most frequently documented racial category being White (2228/3100, 71.8%), followed by Hispanic (320/3100, 10.2%), African American (265/3100, 8.6%), other/unknown (256/3100, 8.1%), and Asian (41/3100, 1.3%). Baseline comparability of all feature variables was assessed using chi-square or Fisher’s exact tests, with statistical significance set at a two-sided p-value < 0.05. All analyses were conducted using SPSS software, and the Benjamini-Hochberg(BH) method was employed to adjust p-values for multiple comparisons, thereby enhancing statistical rigor.

**Table 1:** Baseline values of clinical features based on local databases incorporated into the model.

Feature	Total (*N* = 250)	Phenotype A (*n* = 47)	Phenotype B (*n* = 109)	Phenotype C (*n* = 94)	*P*-value^1^
HR	115.7 (30,220)	109.4 (57,160)	114 (30,168)	119.3 (30,220)	0.0015
SBP	103.7 (37,199)	128.3 (59,199)	101.9 (51,181)	91.9 (37,162)	0.0015
Breath	25.2 (3,76)	20 (12,33)	24 (7,76)	25 (4,42)	0.004
TEMP	37.2 (34,41)	37.2 (34,40)	37.1 (34,40)	37.4 (36,41)	0.4
NA	138.8 (112.6,173.6)	138.8 (120,169)	137.8 (112.6,173)	140.2 (119.1,173.6)	0.048
HB	104.8 (33,213)	113.9 (63,213)	96.8 (33,171)	111.4 (47,170)	0.0015
BUN	17.9 (3,96)	17.3 (3,96)	13.8 (3,81)	21 (3.8,89)	0.0015
CRP	131.7 (2,415.9)	98.3 (2,229.4)	74.1 (2.3,185.1)	215.2 (81,415.9)	0.0015
PaO2/Fi O2	228.1 (30,665)	395.7 (221,665)	157.7 (30,840)	225.9 (31,460)	0.0015
WBC	12.7 (0.5,56.5)	9.8 (0.45,31.6)	10.1 (1.2,34.3)	18.7 (1.1,56.5)	0.0015
FIB	4.53 (1,19)	4.6 (1.3,10)	4.3 (1,19)	4.8 (1,9.4)	0.0015
TBIL	27.3 (3,185.7)	25.5 (3,159.4)	32.8 (3,185.7)	23.5 (4,90.7)	0.07

^1^The *p*-values of the statistical analysis were corrected using the Benjamini-Hochberg method for multiple comparisons.

**Table 2:** External validation of baseline values of clinical features incorporated into the model by the eICU database.

Feature	Total (*N* = 3100)	PhenotypeA (*n* = 528)	PhenotypeB (*n* = 762)	PhenotypeC (*n* = 1810)	*P*-value
HR	116 (40,220)	116 (40,220)	103 (40,198)	120 (40,216)	0.0017
Breath	31.3 (5,60)	32 (5,60)	9 (5,30)	36 (20,60)	0.0017
SBP	110 (50,220)	108 (50,203)	100 (50,199)	120 (55,240)	0.0017
TEMP	36.4 (34,41.6)	36.4 (34.3,40.5)	36.5 (34,40.3)	37.1 (35,41.6)	0.008
NA	136 (110,170)	136 (117,157)	137 (110,162)	136 (115,170)	0.012
HB	99 (30,188)	98 (38.5,157)	98 (41,177)	99 (30,188)	0.43
BUN	35 (9,229)	23 (10,142)	25 (9,150)	27 (10,229)	0.0017
WBC	11.6 (0.2,149.1)	11.15 (0.5,79.6)	10.9 (0.5,126.6)	12 (0.2,149.1)	0.003
FIB	4.2 (1,10.5)	4.5 (2.1,9.1)	3.6 (1.2,8.7)	4.2 (1,10.5)	0.0017
TBIL	7.4 (0.1,300)	7 (0.78,195)	8 (0.2,300)	7 (0.1,300)	0.0017
CRP	53 (3,421)	41.79 (3.7,252)	53.2 (3,218)	56.6 (7,421)	0.02
PaO2/FiO2	230 (40,981.4)	598.5 (360.7,981.4)	234.1 (80,700.9)	182.5 (80,484.3)	0.0017

### Derivation of sepsis sub-phenotypes

**Fig. 2:**
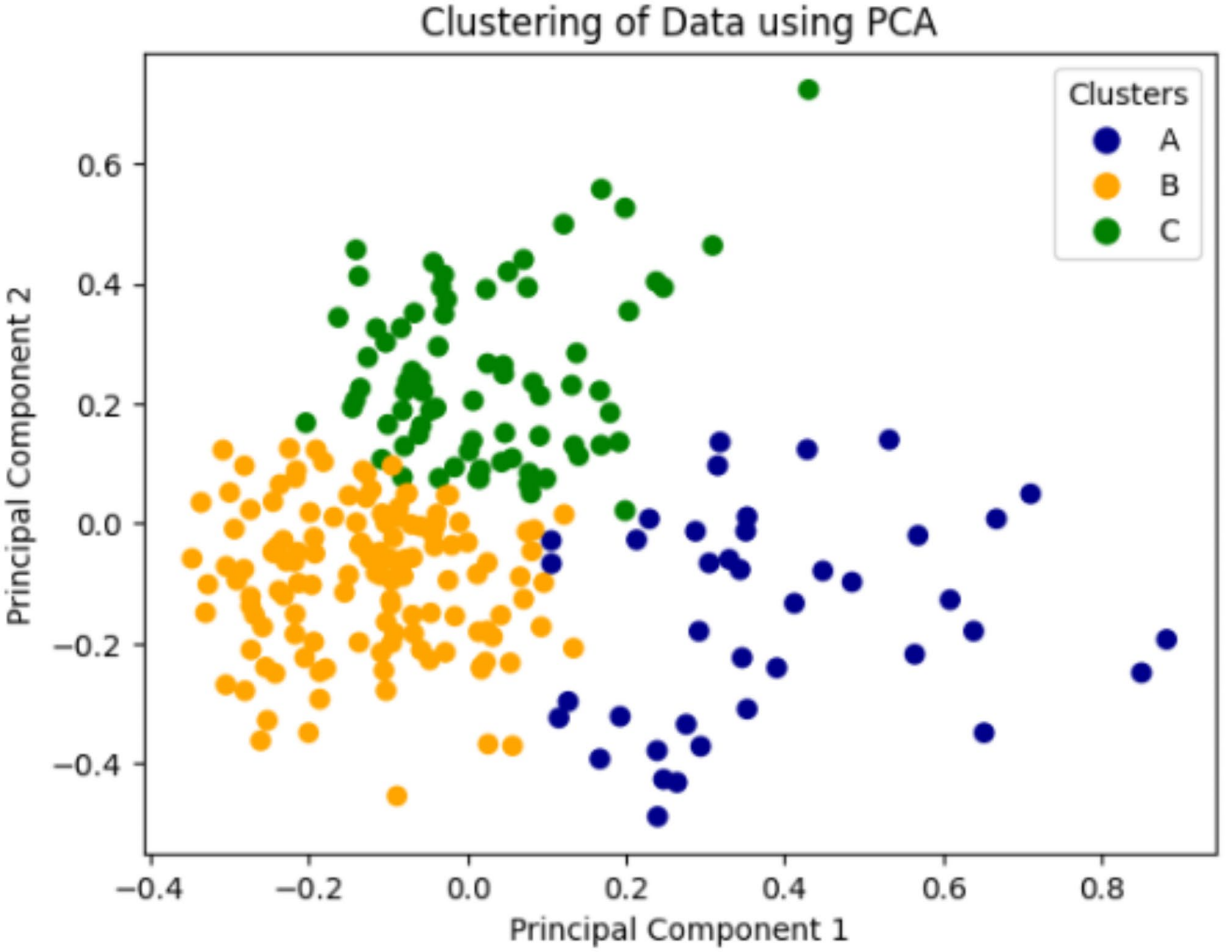
Principal component analysis visualization clustering results. Blue : phenotype A; Green : phenotype B; Yellow : phenotype C.

A correlation analysis of the included features was performed considering the corresponding Pearson correlation coefficients. It was observed that there was no high correlation between the features included in the model. To ensure the robustness of the results, this study performed a sensitivity analysis on the clustering outcomes by testing K-values ranging from 2 to 8. The Silhouette scores varied between 0.12 and 0.26. The optimal clustering was achieved at k = 3, with a Silhouette score of 0.26, a Calinski-Harabasz score of 135.4, and a Davies-Bouldin score of 1.2. To further validate this choice, two clinical experts from Anhui University of Medical Sciences assessed the results and confirmed the appropriateness of selecting k = 3. To mitigate the limitations posed by the size of the internal validation dataset and to enhance the generalizability of the findings. the same K-Means clustering evaluation was conducted using the eICU database. The Silhouette score and the Calinski-Harabasz score are both at high levels. Therefore, the three-category model provided the best fit for that study. For ease of memorization, the three categories were hereafter referred to as phenotypes A, B, and C. A principal component analysis (PCA) of the results was used to create a two-dimensional image to demonstrate the disparities after categorization to better visualize the three phenotypes. Figure 2 is a two-dimensional image of the local dataset produced using PCA to show the differences after sepsis staging.

**Fig. 3:**
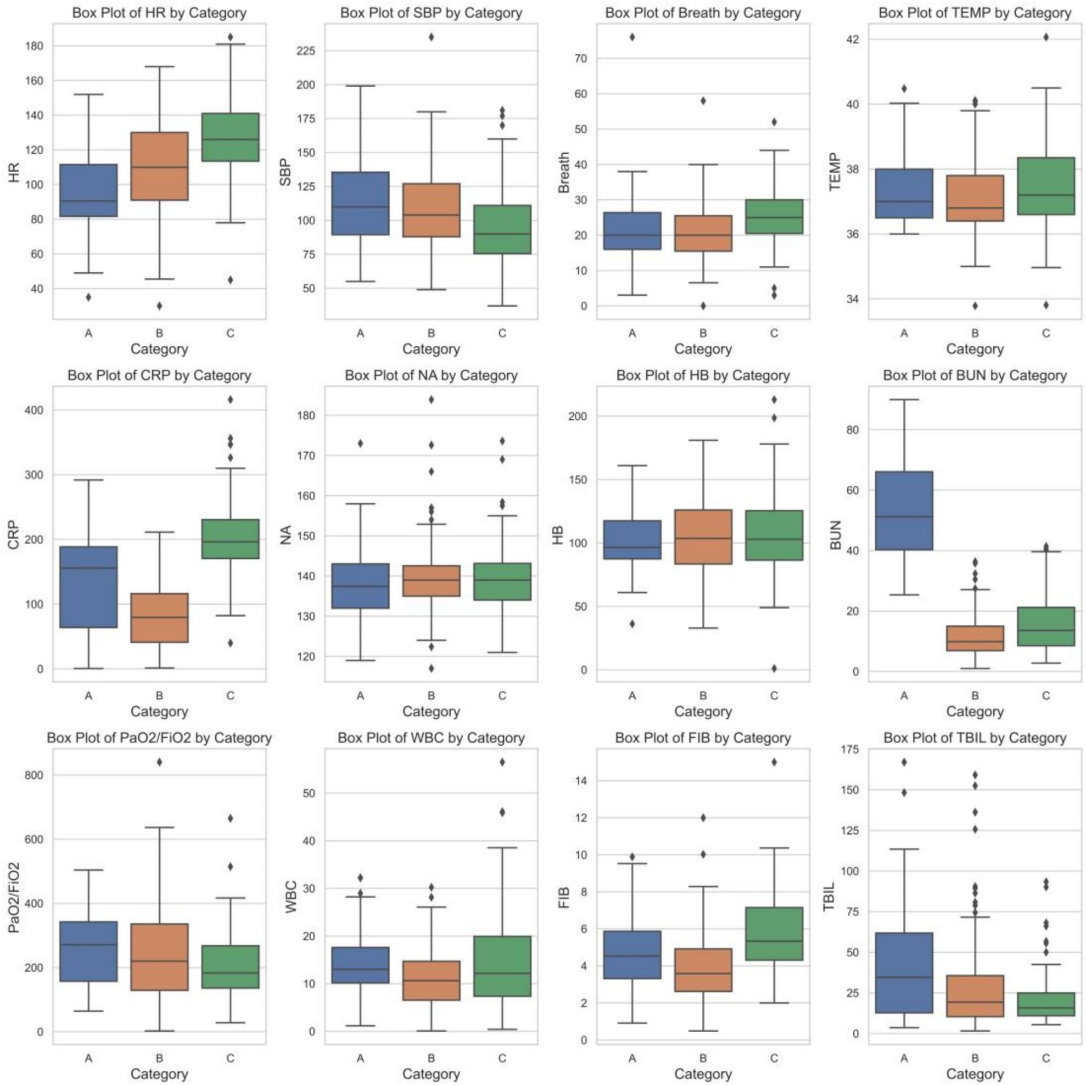
Differences and comparisons among the three phenotypes obtained after clustering in patients with sepsis.

Figure 3 shows the specific details of the variables that feature the three phenotypes. It was observed that patients in group A (47 patients, 18.8%) exhibited elevated SBP, rapidly increasing BUN, and increased TBIL levels, and group B (109 patients, 43.6%) exhibited increased HR and decreased BUN and CRP levels. In contrast, group C (94 patients, 37.6%) showed a rapid increase in HR, decreased SBP, increased TEMP, CRP, FIB, and WBC levels, and decreased PaO2/FiO2 and BUN levels. Regarding outcomes, patients with phenotype A exhibit markedly elevated levels of urea nitrogen, more pronounced renal impairment, and the presence of inflammatory symptoms, thus necessitating a treatment regimen that prioritizes the improvement of renal function. In contrast, patients with phenotype C typically exhibit a more severe inflammatory response and multiorgan dysfunction. Disturbances in biomarkers, such as elevated C-reactive protein (CRP) levels and decreased PaO2/FiO2 ratios, are key indicators of poor clinical performance and contribute to the higher mortality rate observed in phenotype C.

### Phenotypic reproducibility and clinical outcome analysis

**Fig. 4:**
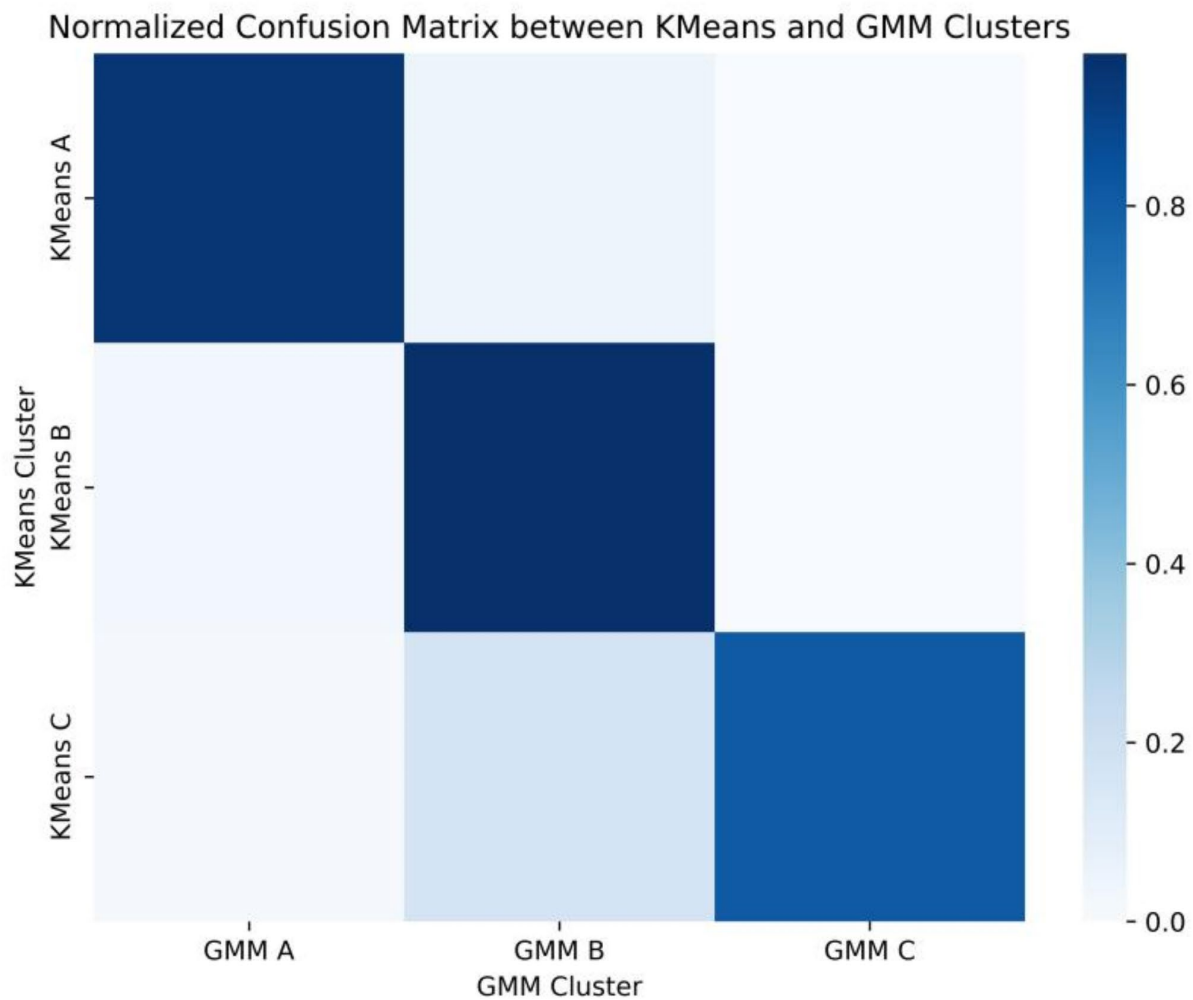
Normalized confusion matrix was constructed to compare K-Means and GMM clustering results. Correlation analysis was conducted between the outcomes of K-Means clustering classes A, B, and C, and those of GMM clustering classes A, B, and C.

Gaussian Mixture Models (GMM) were employed to replicate phenotypes, and latent class analysis validated a statistically adequate fit for the three-class model. Reanalysis of the local sepsis dataset using GMM yielded results closely aligning with the three patient phenotypes identified via KMeans clustering. This alignment is visually demonstrated in Fig. 4 through a confusion matrix, highlighting the high concordance between clusters derived from both algorithms. Consistent outcomes were also observed when GMM was applied to phenotype data from the eICU dataset.

**Fig. 5:**
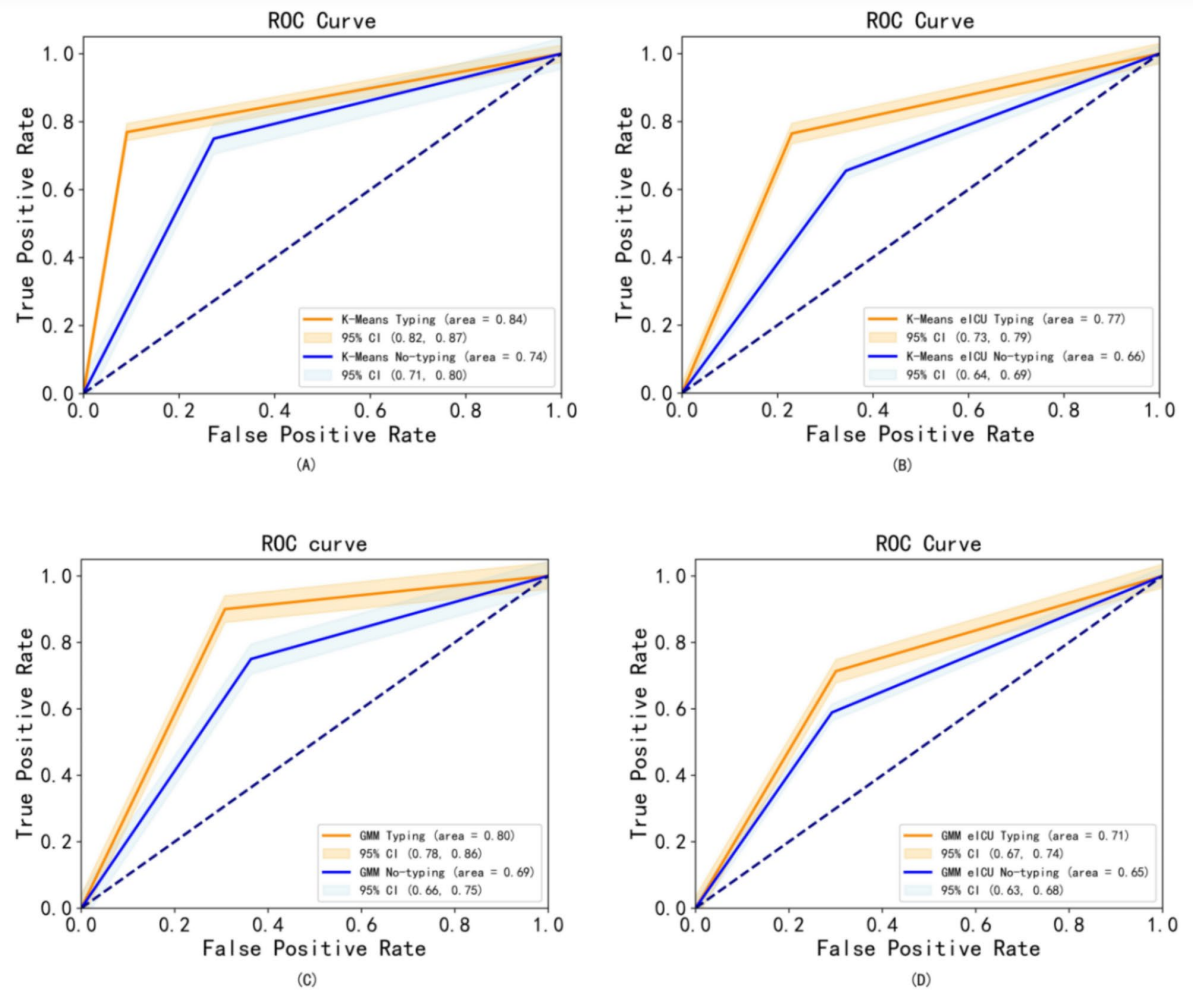
The ROC curves for K-Means clustering (panels **A** and **B**) and GMM clustering (panels **C** and **D**). The curves compare the prediction of patient clinical outcomes using models constructed from both local and eICU datasets, with results shown for both typed (clustered) and untyped (non-clustered) data.

To robustly assess the impact of sepsis phenotypes on the prediction of clinical outcomes, the study was internally validated using data from Anhui Province and externally validated using the eICU database. The validation methods were consistent, using the same feature set and multiple imputation techniques for missing clinical data. After preprocessing, the data were clustered and logistic regression models were trained to predict 28-day clinical outcomes based on assigned phenotypes. These predictions were compared with the results for non-clustered data. To compare the clustering methods, the study analyzed the effect of K-Means and GMM clustering on clinical outcomes, and the results are shown in Fig. 5. The results of the internal and external validation model tests showed that the model trained based on the K-Means clustering results demonstrated higher accuracy in predicting clinical outcomes. The internal validation model had an F1 score of 0.82, a recall of 0.76, and an AUC of 0.84 (95% CI 0.82–0.87), up from 0.74 (95% CI 0.71–0.80). External validation also improved significantly, with an AUC of 0.77 (95% CI 0.73–0.79) from 0.66 (95% CI 0.64–0.69). To offer a more comprehensive evaluation of the model, this study incorporated additional performance metrics such as sensitivity and specificity. As evidenced in Table 3, these metrics demonstrated substantial improvements in both internal and external validation, thereby underscoring the model’s robustness.

**Table 3:** Presents the scores of the model evaluation using the logistic algorithm for the local dataset and the eICU dataset.

Source	Model	Accuracy	Precision	Recall	F1	Sensitivity	Specificity	AUC
K-Means (Local)	Logistic (type/No type)	**0.84**/0.74	**0.90**/0.75	**0.76**/0.71	**0.82**/0.74	**0.79**/0.75	**0.88**/0.73	**0.84**/0.74
K-Means (eICU)	Logistic (type/No type)	**0.78**/0.70	**0.72**/0.73	**0.78**/0.70	**0.72**/0.71	**0.77**/0.66	**0.71**/0.67	**0.77**/0.66
GMM\(Local)	Logistic (type/No type)	**0.78**/0.65	**0.70**/0.77	**0.83**/0.65	**0.77**/0.72	**0.84**/0.67	**0.69**/0.66	**0.80**/0.69
GMM (eICU)	Logistic (type/No type)	**0.73**/0.65	**0.70**/0.67	**0.72**/0.64	**0.71**/0.66	**0.72**/0.64	**0.71**/0.66	**0.71**/0.65

It includes a comparative analysis of the performance of classifiers trained on phenotypes derived from K-Means and GMM clustering, juxtaposed against those trained using traditional non-clustering methods.

### Feature selection evaluation

**Fig. 6:**
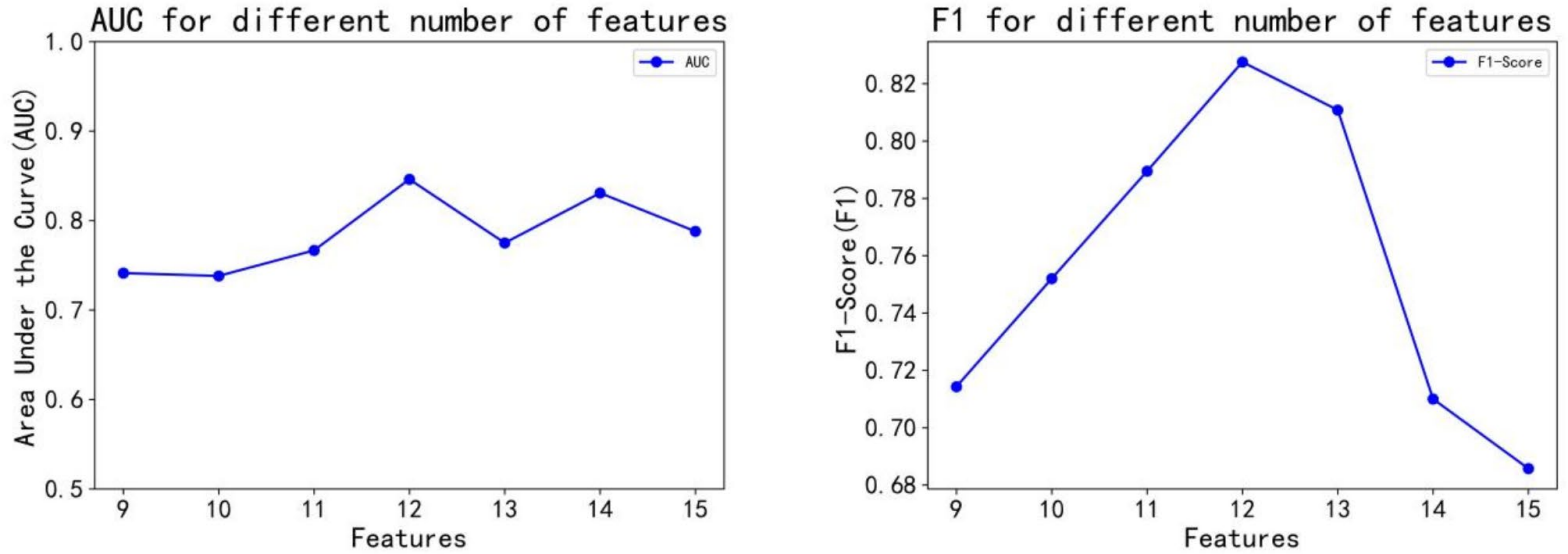
Line plot depicting model F1 and AUC based on varying numbers of features, illustrating specific trends observed from 9 to 15 features.

To validate the selection of the 12 features used in the study, the researchers removed the three features with the highest p-values—TEMP, NA, and TBIL—and compared models using sets of 9, 10, and 11 features. Additionally, to perform a sensitivity analysis comparing features with more than 40% missing values and a p-value of less than 0.05 for statistical analysis, creatinine, lactate, and oxygen saturation were introduced as features 13, 14, and 15. Analysis of the F1 scores and AUC results indicated that the model with all 12 features performed optimally. From the results, it is confirmed that too many missing values have a large impact on the model. Figure 6 presents a line graph showing F1 and AUC outcomes across the different feature sets, confirming that the initial selection of 12 features was the most effective.

## Discussion and conclusion

Unsupervised cluster analysis was widely used for disease classification, and the K-Means clustering algorithm was the most common unsupervised clustering algorithm. To date, in disease classification, researchers successfully used the K-Means clustering algorithm to identify Parkinson’s disease phenotype, COPD phenotype, and heart disease phenotype^21–23^. However, it was still tricky to select practical features to apply the research to cluster analysis of sepsis, which was highly related to the diverse symptoms of sepsis and the reliance on physicians’ experience for diagnosis^24,25^. Therefore, in this study, we started from routine clinical data and utilized K-Means algorithm cluster analysis to identify sepsis phenotypes accurately. By capturing 12 features for data clustering, significant differences were observed among the three phenotypes. Notably, phenotype C exhibited the most critical clinical abnormalities, including increased heart rate, decreased systolic blood pressure, elevated levels of CRP and FIB, as well as reduced PaO2/FiO2 and BUN. These findings align with previous research, indicating that increases or decreases in CRP and PaO2/FiO2 are critical indicators of poor clinical outcomes^17,19^. CRP is a marker of infection, with higher values indicating more severe illness, while PaO2/FiO2 assesses respiratory function^26,27^. Phenotype-driven models help physicians risk stratify patients at an early stage by identifying patients at high risk, e.g., patients with phenotype C in terms of clinical presentation typically have a more severe inflammatory response and multiorgan dysfunction, requiring aggravated anti-inflammatory therapy as well as comprehensive organ supportive therapy, and provide more medical attention to patients with phenotype C. The mortality rate in phenotype C was approximately 56.1%, higher than 51.3% in phenotype A and 46.7% in phenotype B, highlighting a higher mortality rate in phenotype C than in A and B. These results underscore the importance of sepsis phenotypes in predicting clinical outcomes.

Several studies have shown that there were significant problems in the classification of sepsis^28–30^. The first issue was the features used for classification, most commonly SOFA scores and some protease-like substances, which lacked sensitivity and were too expensive for mass dissemination^3^. The second sepsis, being a heterogeneous disease, relies heavily on the clinician’s diagnosis and is inherently subjective. The third issue was that the majority of sepsis cases in the world occurred in low- and middle-income countries. Still, the MIMIC and eICU data utilized were from high-income Western countries. The fourth issue is that most clinical outcomes are predicted using clinical features, but differences in sepsis severity and differences in heterogeneity can affect clinical outcomes. Therefore, in this study, routine clinical data were collected, and patients were clustered and analyzed using the K-Means clustering algorithm to train classifiers for each category of patients to capture trends in patient features for specific categories, which is more effective compared to traditional non-clustering models for clinical outcome prediction. This was similarly validated during the external validation process.

This study has several limitations. First, the data used in this study were obtained from critically ill patients in the ICU. Since ICU patients often have more complex clinical presentations, the generalizability of the study’s findings is limited. To improve the model’s applicability, future research should include data from patients non-ICU. Second, while we compared the baseline demographic characteristics of our local dataset with those of the eICU dataset, differences in ethnicity and institutional clinical characteristics may introduce inherent biases in the data, potentially affecting the model’s generalizability. Third, In this study, only common data features were included for cluster analysis. However, incorporating additional parameters such as metabolomics and imaging data could potentially enhance the outcomes of patient cluster analysis. This possibility warrants further investigation in future research. Lastly, we acknowledge that while clustering analysis provides an in-depth understanding of the heterogeneity of sepsis, the direct link between these phenotypes and clinical treatment outcomes still requires further investigation. Future research endeavors should focus on integrating a broader spectrum of data sources, executing multicenter data collaborations, and devising prospective cohort studies to robustly validate the impact of phenotype-driven approaches on clinical outcomes.

### Summary

The clinical phenotype of patients with sepsis could be rapidly determined by K-Means cluster analysis using common clinical data. This study successfully identified and validated three distinct sepsis phenotypes. The study results demonstrated that the classifier built on these phenotypes significantly outperformed traditional methods in predicting patients’ 28-day clinical outcomes using clinical features alone. This improved accuracy could aid clinicians in enhancing treatment outcomes and reducing mortality rates. However, further studies were needed to confirm the effectiveness of these phenotypes in clinical practice.

### Data analysis

Data extraction was performed using Navicat Premium software (version 15.0). The extracted data were then subjected to statistical analysis using IBM SPSS Statistics software (version 25.0). To control for multiple comparisons, p-values were adjusted using the Benjamini-Hochberg (BH) method. Additionally, detailed algorithmic studies were conducted using Jupyter Notebook (version 6.4.12).

## Data Availability

The original contributions presented in this study are included in the article, further inquiries can be directed to the corresponding author/s.

## References

[1] Verdonk, F., Blet, A. & Mebazaa, A. The new sepsis definition. *Curr. Opin. Anaesthesiol.* **30** (2), 200–204. 10.1097/aco.0000000000000446 (2017). 28207566 10.1097/ACO.0000000000000446

[2] Shankar-Hari, M. et al. Developing a new definition and assessing new clinical criteria for septic shock. *Jama* **315** (8). 10.1001/jama.2016.0289 (2016). 10.1001/jama.2016.0289 PMC4910392 26903336

[3] Singer, M. et al. The third international consensus definitions for sepsis and septic shock (Sepsis-3). *JAMA* **315** (8), 801–810. 10.1001/jama.2016.0287 (2016). 26903338 10.1001/jama.2016.0287 PMC4968574

[4] Peach, B. C. Implications of the new sepsis definition on research and practice. *J. Crit. Care* **38**, 259–262. 10.1016/j.jcrc.2016.11.032 (2017). 28011419 10.1016/j.jcrc.2016.11.032

[5] Luyao Zhou, M., Shao, C., Wang, Y. & Wang An early sepsis prediction model utilizing machine learning and unbalanced data processing in a clinical context. *Reventive Med. Rep.* **102841**, 2211–3355. 10.1016/j.pmedr.2024.102841 (2024). 10.1016/j.pmedr.2024.102841 PMC11345914 39188971

[6] Wang, H. E. et al. National variation in United States sepsis mortality: a descriptive study. *Int. J. Health Geogr.* **9**, 9. 10.1186/1476-072X-9-9 (2010). 20156361 10.1186/1476-072X-9-9 PMC2831852

[7] Caraballo, C. & Jaimes, F. Organ dysfunction in sepsis: an ominous trajectory from infection to death. *Yale J. Biol. Med.* **92** (4), 629–640 (2019). 31866778 PMC6913810

[8] Adhikari, N. K. J. et al. Critical care and the global burden of critical illness in adults. *Lancet* **376** (9749), 1339–1346 (2010). 20934212 10.1016/S0140-6736(10)60446-1 PMC7136988

[9] Kong, G., Lin, K. & Hu, Y. Using machine learning methods to predict in-hospital mortality of sepsis patients in the ICU. *BMC Med. Inf. Decis. Mak.* **20**, 251. 10.1186/s12911-020-01271-2 (2020). 10.1186/s12911-020-01271-2 PMC7531110 33008381

[10] Bao, C. & Deng, F. Zhao, Machine-learning models for prediction of sepsis patients mortality. *Med. Intensiva* 2173–5727. 10.1016/j.medine.2022.06.024 (2023). 10.1016/j.medine.2022.06.024 36344339

[11] Donnelly, J. P. et al. Application of the third international consensus definitions for sepsis (Sepsis-3) classification: a retrospective population-based cohort study. *Lancet Infect. Dis.* **17** (6), 661–670 (2017). 28268067 10.1016/S1473-3099(17)30117-2 PMC5449202

[12] DeMerle, K. M. et al. Sepsis subclasses: A framework for development and interpretation. *Crit. Care Med.* **49** (5), 748–759. 10.1097/CCM.0000000000004842 (2021). 33591001 10.1097/CCM.0000000000004842 PMC8627188

[13] Xu, Z. et al. Sepsis subphenotyping based on organ dysfunction trajectory. *Crit. Care* **26**, 197. 10.1186/s13054-022-04071-4 (2022). 35786445 10.1186/s13054-022-04071-4 PMC9250715

[14] Bhavani, S. V. et al. Identifying novel sepsis phenotypes using temperature trajectories. *Am. J. Respir Crit. Care Med.* **200** (3), 327–335 (2019). 30789749 10.1164/rccm.201806-1197OC PMC6680307

[15] Soussi, S. et al. CCCTBG trans-trial group study for InFACT - the international forum for acute care trialists. Identifying clinical subtypes in sepsis-survivors with different one-year outcomes: a secondary latent class analysis of the FROG-ICU cohort. *Crit. Care* **26** (1), 114. 10.1186/s13054-022-03972-8 (2022). 35449071 10.1186/s13054-022-03972-8 PMC9022336

[16] Pollard, T. J. et al. The eICU collaborative research database, a freely available multi-center database for critical care research. *Sci. Data*10.1038/sdata.2018.178 (2018). 30204154 10.1038/sdata.2018.178 PMC6132188

[17] Hu, C. et al. Application of machine learning for clinical subphenotype identification in sepsis. *Infect. Dis. Ther.* **11**, 1949–1964. 10.1007/s40121-022-00684-y (2022). 36006560 10.1007/s40121-022-00684-y PMC9617989

[18] Zhang, Z. et al. Identification of subclasses of sepsis that showed different clinical outcomes and responses to amount of fluid resuscitation: a latent profile analysis. *Crit. Care* **22**, 347. 10.1186/s13054-018-2279-3 (2018). 30563548 10.1186/s13054-018-2279-3 PMC6299613

[19] Seymour, C. W. et al. Derivation, validation, and potential treatment implications of novel clinical phenotypes for sepsis. *JAMA* **321** (20), 2003–2017. 10.1001/jama.2019.5791 (2019). 31104070 10.1001/jama.2019.5791 PMC6537818

[20] World Medical Association. World medical association declaration of Helsinki: ethical principles for medical research involving human subjects. *JAMA* **310** (20), 2191–2194. 10.1001/jama.2013.281053 (2013). 24141714 10.1001/jama.2013.281053

[21] Galvin, J. E., Pollack, J. & Morris, J. C. Clinical phenotype of Parkinson disease dementia. *Neurology* **67** (9), 1605–1611. 10.1212/01.wnl.0000242630.52203.8 (2006). 17101891 10.1212/01.wnl.0000242630.52203.8f

[22] Hirai, K. et al. A clustering approach to identify and characterize the asthma and chronic obstructive pulmonary disease overlap phenotype. *Clin. Exp. Allergy* **47** (11), 1374–1382. 10.1111/cea.12970 (2017). 10.1111/cea.12970 28658564

[23] Silva, I. S. et al. Polycystic ovary syndrome: clinical and laboratory variables related to new phenotypes using machine-learning models. *J. Endocrinol. Invest.* **45**, 497–505. 10.1007/s40618-021-01672-8 (2022). 34524677 10.1007/s40618-021-01672-8

[24] DeMerle, K. M. et al. Sepsis subclasses: a framework for development and interpretation. *Crit. Care Med.* **49** (5), 748–759. 10.1097/CCM.0000000000004842 (2021). 33591001 10.1097/CCM.0000000000004842 PMC8627188

[25] Komorowski, M. et al. Sepsis biomarkers and diagnostic tools with a focus on machine learning. *EBioMedicine* 86. 10.1016/j.ebiom.2022.104394 (2022). 10.1016/j.ebiom.2022.104394 PMC9783125 36470834

[26] Terry, W. & Du Clos function of C-reactive protein. *Ann. Med.* **32** (4), 274–278. 10.3109/07853890009011772 (2000). 10852144 10.3109/07853890009011772

[27] Bi, H. et al. The PaO2/FiO2 is independently associated with 28-day mortality in patients with sepsis: a retrospective analysis from MIMIC-IV database. *BMC Pulm Med.* **23**, 187. 10.1186/s12890-023-02491-8 (2023). 37245013 10.1186/s12890-023-02491-8 PMC10225083

[28] Klein Klouwenberg, P. M. C. et al. Classification of sepsis, severe sepsis and septic shock: the impact of minor variations in data capture and definition of SIRS criteria. *Intensive Care Med.* **38**, 811–819. 10.1007/s00134-012-2549-5 (2012). 22476449 10.1007/s00134-012-2549-5

[29] Li, H. et al. Methods for phenotyping adult patients in sepsis and septic shock: A scoping review. *Crit. Care Explor.* **4** (4), e0672. 10.1097/CCE.0000000000000672 (2022). 10.1097/CCE.0000000000000672 PMC8970078 35372844

[30] Papathanakos, G. et al. Clinical sepsis phenotypes in critically ill patients. *Microorganisms* **11** (9), 2165. 10.3390/microorganisms11092165 (2023). 37764009 10.3390/microorganisms11092165 PMC10538192

